# Specialized Pro-Resolving Lipid Mediators: The Future of Chronic Pain Therapy?

**DOI:** 10.3390/ijms221910370

**Published:** 2021-09-26

**Authors:** Mervin Chávez-Castillo, Ángel Ortega, Lorena Cudris-Torres, Pablo Duran, Milagros Rojas, Alexander Manzano, Bermary Garrido, Juan Salazar, Aljadis Silva, Diana Marcela Rojas-Gomez, Juan B. De Sanctis, Valmore Bermúdez

**Affiliations:** 1 Endocrine and Metabolic Diseases Research Center, School of Medicine, University of Zulia, Maracaibo 4004, Venezuela; mervinch12@gmail.com (M.C.-C.); angelort94@gmail.com (Á.O.); pabloduran1998@gmail.com (P.D.); migarocafi@gmail.com (M.R.); amanzano_8@hotmail.com (A.M.); bermarygarrido@gmail.com (B.G.); juanjsv18@hotmail.com (J.S.); aljadisenrique@gmail.com (A.S.); 2 Programa de Psicología, Fundación Universitaria del Área Andina sede Valledupar, Valledupar 200001, Colombia; lcudris@areandina.edu.co; 3 Escuela de Nutrición y Dietética, Facultad de Medicina, Universidad Andres Bello, Santiago 8370035, Chile; diana.rojas@unab.cl; 4 Institute of Molecular and Translational Medicine, Palacký University Olomouc, 77900 Olomouc, Czech Republic; sanctisj@gmail.com; 5 Facultad de Ciencias de la Salud, Universidad Simón Bolívar, Barranquilla 080002, Colombia

**Keywords:** chronic pain, specialized pro-resolving lipid mediators, inflammation, long-term potentiation, central nervous system sensitization, polyunsaturated fatty acids, eicosanoids, nociception, omega 3 fatty acids, pain management

## Abstract

Chronic pain (CP) is a severe clinical entity with devastating physical and emotional consequences for patients, which can occur in a myriad of diseases. Often, conventional treatment approaches appear to be insufficient for its management. Moreover, considering the adverse effects of traditional analgesic treatments, specialized pro-resolving lipid mediators (SPMs) have emerged as a promising alternative for CP. These include various bioactive molecules such as resolvins, maresins, and protectins, derived from ω-3 polyunsaturated fatty acids (PUFAs); and lipoxins, produced from ω-6 PUFAs. Indeed, SPMs have been demonstrated to play a central role in the regulation and resolution of the inflammation associated with CP. Furthermore, these molecules can modulate neuroinflammation and thus inhibit central and peripheral sensitizations, as well as long-term potentiation, via immunomodulation and regulation of nociceptor activity and neuronal pathways. In this context, preclinical and clinical studies have evidenced that the use of SPMs is beneficial in CP-related disorders, including rheumatic diseases, migraine, neuropathies, and others. This review integrates current preclinical and clinical knowledge on the role of SPMs as a potential therapeutic tool for the management of patients with CP.

## 1. Introduction

Chronic pain (CP) is one of the most frequent and difficult-to-manage clinical entities in medical practice [1]. Multiple disorders featuring CP are the leading causes of disability worldwide, corresponding to a significant public health issues [2,3,4]; as well as marked reductions in quality of life related to restrictions of mobility and daily activities, anxiety, and depression [5,6]. Current pharmacological options for the treatment of CP are imperfect, including significant efficacy and tolerability issues [7]. In particular, opioid abuse has been linked to increasingly larger mortality rates in recent years, representing a large-scale epidemic [7,8].

These problems have invigorated neuropharmacological research on CP [9], centering on neuroinflammation as a therapeutic target [10]. Inflammation as a pathophysiologic component of pain has long been recognized in neural phenomena such as peripheral sensitization (PS), central sensitization (CS), and long-term spinal potentiation (LTP) [11]. Recent preclinical and clinical studies have described anti-nociceptive effects for specialized pro-resolving lipid mediators (SPMs), which derive from polyunsaturated fatty acids (PUFA) [12,13,14,15]. These molecules are important regulators of the balance between pro-inflammatory and anti-inflammatory substances, in addition, they might regulate the excessive sensitization of nociceptors after inhibiting specialized channels and thus achieving the suppression of pain [13]. In this way, SPMs would function as a bridge between the immune and nervous systems, and could be the future of CP therapy. The objective of this review is to describe the molecular pharmacological mechanisms through which SPMs act in CP, as well as summarize current preclinical and clinical evidence on SPMs as analgesic agents, serving as a novel approach to CP management.

## 2. Materials and Methods

This is a narrative review in which an extensive literature search was performed on Scopus, EMBASE, PubMed, ISI Web of Science, and Google Scholar databases, from inception to August 2021. The terms “Chronic pain”, “Neuroinflammation”, “specialized pro-resolving lipid mediators and chronic pain”, and “Chronic pain and nociception” were among the ones used throughout the search.

## 3. Results

### 3.1. Specialized Pro-Resolving Lipids Mediators in Pain: The Molecular Basis

**Figure 1:**
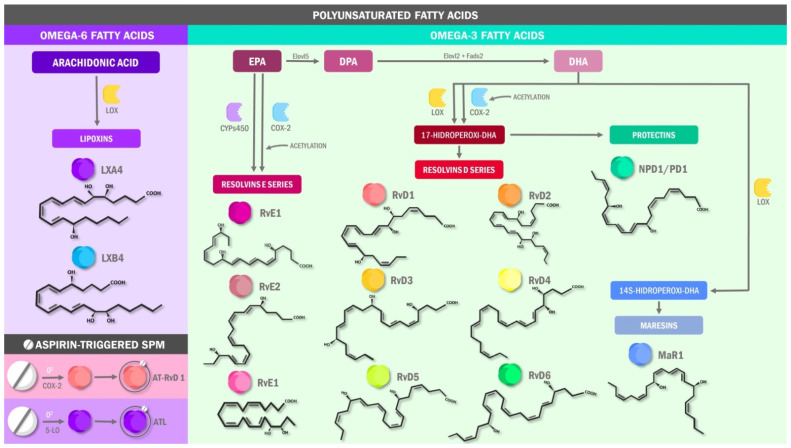
Metabolism of specialized pro-resolving lipid mediators. EPA: eicosapentaenoic acid; DPA: docosapentaenoic acid; DHA: docosahexaenoic acid; RvE1: resolvins E1; RvE2: resolvins E2; RvE3: resolvins E3; RvD1: resolvins D1; RvD2: resolvins D2; RvD3: resolvins D3; RvD4: resolvins D4; RvD5: resolvins D5; RvD6: resolvins D6; NPD1: neuroprotectins 1; MaR1: maresins 1; LXA4: lipoxins A4; AT-RvD: aspirin-triggered resolvin D1; ATL: aspirin-triggered lipoxins; LOX: lipoxygenases; COX-2: cyclooxygenase 2; CYP: cytochromes P450 enzymes.

SPMs are synthesized in an active metabolic process in the latter stages of inflammation, acting as a regulatory mechanism, decreasing pain caused after sensitization of nociceptors, and limiting local tissue damage caused by the inflammatory response [11]. A variety of PUFA are well-recognized substrates for SPM synthesis, including both ω-6 fatty acids such as arachidonic acid (AA), and ω-3 fatty acids such as eicosapentaenoic (EPA), docosahexaenoic (DHA), and docosapentaenoic acids (DPA) [16,17]. Thus, after activation of intracellular phospholipases, these molecules can be used as the initial substrate in the SPMs synthesis pathways, yielding products in two families: Lipoxins (LX), derived from AA, and protectins (PD), maresins (MaR), and resolvins (Rv), derived from the ω-3 fatty acids [18] (Figure 1). The latter may be further categorized into two groups: E-series (RvE) and D-series (RvD) resolvins. RvE synthesis involves cyclooxygenase-2 activity, as well as processing by lipoxygenases such as 5-lipoxygenase (5-LOX), which forms RvE1.

Alternatively, RvD may be produced from DHA via 5-LOX and 15-lipooxygenase (15-LOX) to form 17S-hydroperoxy-DHA, which can finally be converted to RvD1, RvD2, RvD3, and RvD4. Interestingly, acetylsalicylic acid (AAS) appears to induce the synthesis of RvD and LX by acetylating COX-2 and thereby changing its enzymatic properties [19,20]. DHA may also be a precursor of PD and MaR [21], which are abundant in murine and human neurons and macrophages, respectively [22,23]. Despite the close relationship between PUFAs, SPMs and inflammatory pathology improvements, the mechanism in which COX-2 acetylation is involved continues to be discussed nowadays. 

Furthermore, the synthesis of SPMs is key for promoting an anti-inflammatory and pro-resolution state, and by extension, relieving peripheral inflammatory pain [17]. This change from a pro-inflammatory to an anti-inflammatory state appears to hinge on a phenotypic modification in 15-LOX functionality induced by a PGE2 peak, which propels a shift from the production of leukotriene B4 (LTB4) to LX, leading to Rv, PD, and MaR synthesis [24,25]. This process is driven by increased translation of key enzymes for ARNm codification [26,27].

In turn, SPMs activate a variety of receptors in several immune cells, for example, the N-formyl peptide receptor 2 (ALX/FPR2), widely recognized in neutrophils, monocytes, T cells, synovial fibroblasts, and glial cells; whose main ligands are LXA4 and ATL [28]. Moreover, LX have been observed to antagonize pro-inflammatory mediators such as IL-6 and IL-8 in various cells in the respiratory tract and inhibit TNF-α release in human T cells [29]. In polymorphonuclear cells (PMN), LX also activate ALX/FPR2, decrease leukocyte infiltration and inhibit transmigration, adhesion, degranulation, and chemotaxis of neutrophils, as well as the generation of superoxide. Rapid phosphorylation of Lymphocyte-specific protein 1 (LSP1) and polyisoprenyl phosphates is a paramount step in this process [30]. Likewise, LXA4 competes with other ALX/FPR2 ligands such as LTB4, PGE2, and N-formyl-Met-Leu-Phe peptide (fMLP), reducing their activity in PMN [30,31,32,33,34,35]. In addition, by disrupting chemotaxis, SPMs may also indirectly prevent the formation of neutrophil extracellular traps, thus attenuating further recruitment and favoring resolution of inflammation [36].

On the other hand, LX are the main products obtained from AA in this context, via 5-LOX and 15-LOX oxygenation. This yields LXA4 and LXB4, which have been isolated from endothelial cells, leukocytes, and human platelets [37]. Notably, ASA appears to promote the production of 15R-hydroperoxy-eicosatetraenoic acid, a substrate for 5-LOX to form 15-epi-LXA4, an LX epimer. These aspirin-triggered lipoxins (ATL) may have unique and beneficial biological properties in comparison with the effects of other related agents, such as non-steroidal anti-inflammatory drugs (NSAID). Indeed, ATL may correlate to a key role for ASA specifically in the clinical management of inflammatory and CP-related disorders [26,38]. 

The activation of ALX/FPR2 also appears to induce changes in the phosphorylation of cytoskeleton proteins, arresting the cell cycle and preventing phosphorylation in pro-inflammatory pathways, like the activation of nuclear factor kappa B (NF-κB) [30,39]. Moreover, LXA4 can also antagonize CysLT1 receptors in PMN, endothelial and mesangial cells [40]; and inhibit proliferation induced by leukotriene D4 by modulating platelet-derived growth factor receptor (PDGF) transactivation, and therefore, phosphoinositoside 3-kinase (PI3K) activation and the mitogenic response [31]. In neurons, LXA4 signaling can also be potentiated by sphingosine-1-phosphate, which can acetylate neuronal COX-2 to drive a skew towards the production of the powerfully pro-resolutive 15-epi-LXA4, similarly to the events seen in the synthesis of ATL [41]. Lastly, both LXA4 and LXB4 may also inhibit the chemotaxis of neutrophils induced by LTB4, as well as eosinophil degranulation by PDGF [31].

In other cell types, such as monocytes, LX promote the resolution of inflammation, as they are capable of mediating chemotaxis and adhesion without the release of reactive oxygen species (ROS) and degranulation [42]. Furthermore, in macrophages, LXA4 stimulates phagocytosis of apoptotic PMN [12]. This process occurs via activation of small GTPases, with the subsequent redistribution of cytoskeletal proteins for the assembly of cytoplasmic extensions and pseudopods [43]. Meanwhile, in neutrophils, similar changes are mediated by inhibition of protein kinase C-BII (PKCBII). This enzyme promotes inflammation by triggering the conversion of polysoprenyl diphosphate phosphatase 1 into presqualene diphosphate and then presqualene monophosphate, a positive stimulus for several functional cell responses in inflammation [44]. On the other hand, transforming growth factor β (TGF-β) is synthesized during phagocytosis, which actively suppresses the release of pro-inflammatory cytokines [45], and promotes SPMs biosynthesis, further favoring resolution [46]. Finally, activation of ALX/FPR2 can inhibit IL-10 production by B cells and TNF-α in T cells, highlighting the anti-inflammatory role of SPMs in the adaptive immune response, as seen in chronic inflammation [47,48]. 

Other SPMs with notable mechanisms of action include RvD and RvE. Particularly, RvD1 shares the affinity for the ALX/FPR2 receptors with LXA4; and also binds to other GPCR, such as DRV1/GPR3. These receptors are found in PMN, monocytes, macrophages, and endothelial cells. Their activation appears to involve upregulation of specific microRNA such as miR-208 and IL-10 while downregulating miR-219, which modulates 5-LOX and reduces LTB4 levels [21,49]. RvD1 can also reduce actin polymerization and CDb11 activity [50], powerfully promoting leukocyte adhesion, migration, and phagocytosis [51]. Others, like RvD3 and RvD5, also act on these receptors [12]. Another recently discovered receptor, DRV2/GPR18—found in bone marrow, monocytes, and macrophages—is also activated by RvD2, in association with increased phagocytosis via modulation of the protein kinase A (PKA) and STAT3 pathways [52,53]. RvD may also inhibit the synthesis of INF-y and TNF-α in Th1 and Th17 cells, resulting in increased production of Treg cells, lower IL-6 levels, and increased production of IgM and IgE by B cells [54]. RvD1 can inhibit the class change of IgG to IgE by stabilizing regulatory protein BCL-6. These events reflect the role of SPMs in allergic processes [48,55]. 

Interestingly, neuropathic pain in microglia activates by phosphorylation mitogen-activated protein kinase (MAPK), increasing prostaglandin E2 (PGE2), which mediates microglial activation and subsequent pain enhancement, as counter-regulators RvD1 and LXA4 in microglia can inhibit TNF release, an important factor in pain mediation [56]. In addition, RvD1 has functions as a promoter of conjunctival cell health using a different mechanism than the one mentioned before, which is the stimulation of mucin secretion by goblet cells. This is mediated by intracellular calcium increase [57]. In the case of RvD2, it has a similar role but it also increases nitric oxide and prostacyclin production in endothelial cells as well as microbial killing promotion. This results in controlled PMN adhesion, thus acting as an anti-inflammatory substance [58].

Among RvE, RvE1, and RvE2 can bind to ERV1/Chem23 receptors, abundantly expressed in monocytes, and more sparsely on neutrophils, M2 macrophages, and dendritic cells. RvE1 can compete with chemerin, a pro-inflammatory ligand of this receptor, resulting in inhibition of signaling by NF-kB, MAPK/ERK1-2, and PI3K/AKT [59,60], which are essential for inflammation and phagocytosis [12,61]. RvE1 is also a partial agonist of the LTB4 receptor, blocking the calcium-mediated intracellular response induced by this leukotriene in leukocytes, and inhibiting chemotaxis [62,63]. Furthermore, RvE1 is one of the few SPMs that can inhibit cytokine production in Th2 cells [54]. 

On the other hand, MaR activity is prominent in macrophages, where they may be synthesized from DHA [64]. 13S, 14S-epoxide maresin induces the differentiation of pro-inflammatory M1 macrophages to M2 macrophages, which poses anti-inflammatory and pro-resolving properties, releasing PDGF, IL-10, and TGF-β [65]. MaR1 increases phagocytosis and activates PKC isoforms, which limit neutrophil infiltration, decrease IL-6, TNF-α, and chemokine production. It also prevents the activation of NF-κB by inhibiting IkB kinase (IKK), not allowing the dissociation of the inhibitor of NF-κB (IκBα) from NF-kB itself [66,67,68,69]. Even though it is not part of the SPMs family, flavonoids-derivate pharmaceutics, such as Flavocoxid, have been studied as pro-resolving therapeutics that have a similar mechanism to MaR, specifically as a possible inhibitor of NF-kB. It also acts as a dual inhibitor of COX-2 and 5-LOX. Furthermore, it has been associated with increased levels of LXA4 production [70]. Finally, other SPMs may also modulate immune cell functionality and cytokine synthesis: PD appears to inhibit T cell migration and promote their apoptosis in vivo, in association with lower TNF-α levels [71]. It also reduces signaling by NF-kB, expression of COX-2, and infiltration by PMN. PD1 is also a ligand for GPR37, promoting phagocytosis of apoptotic cells in inflammation. However, the underlying mechanisms remain unclear [72,73,74].

### 3.2. Specialized Pro-Resolving Lipids Mediators in the Neurobiology of Pain: Anti-Inflammatory and Analgesic Mechanisms

A large part of the pharmacological interest in SPMs focuses on their capacity for modulation of neuroinflammation, as pain results from the interplay between immune and nervous cells. Historically, the resolution of the inflammatory response was construed as a passive process depending on the spontaneous wane of pro-inflammatory factors. However, at present, active modulation by various immune cells is recognized as a paramount factor in this process [12,75]. SPMs may be the key mediators in this context, as explained before, relieving peripheral inflammatory pain [17]. 

**Figure 2:**
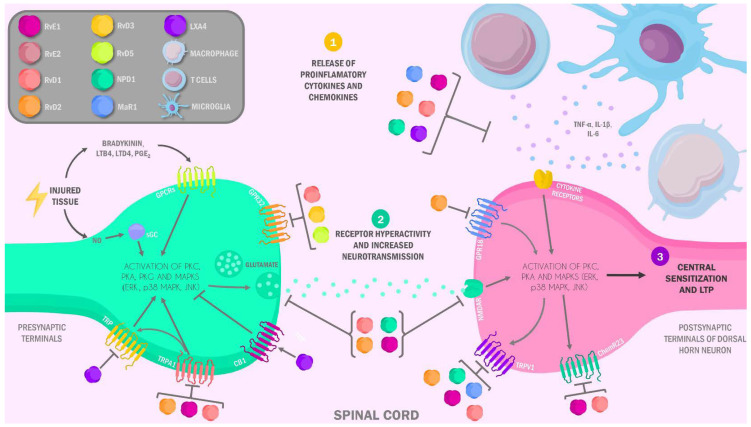
Role of specialized pro-resolving lipid mediators in central sensitization and long-term spinal potentiation in pain. Inflammation caused by local or peripheral injury impacts the nervous system through pro-inflammatory mediators secreted by immune and specialized cells, with the subsequent upregulation and hyperactivity of nociceptors and other receptors related to pain perception, leading to uncontrolled synaptic activity and development of central sensitization and long-term spinal potentiation. RvE1: resolvins E1; RvE2: resolvins E2; RvD1: resolvins D1; RvD2: resolvins D2; RvD3: resolvins D3; RvD5: resolvins D5; NPD1: neuroprotectins 1; MaR1: maresins 1; LXA4: lipoxins A4; LTB4: leukotriene B4; LTD4: leukotriene D4; PGE2: protaglandines E2; GPCRs: G protein-coupled receptors; TRP: transient receptor potential; TRPV1: transient receptor potential vanilloid subtype 1; TRPA1: transient receptor potential cation channel subfamily A member 1; CB1: cannabinoid receptor 1; NMDAR: N-Methy-D-Aspartate Receptor; ChemR23: chemerin receptor 23; LTP: long-term potentiation; TNF-α: Tumoral nuclear factor; IL-1b: Interleucin 1b; IL-6: Interleucin 6; PKC: protein kinase C; PKA: protein kinase A; ERK: extracellular signal-regulated kinase; MAPK: mitogen-activated protein kinases; JNK: JUN N-terminal kinase; NO: nitric oxide; sGC: soluble guanylyl cyclase receptor.

Peripheral inflammatory pain is a consequence of the sensitization of peripheral nociceptors by various signals, such as the recruitment of macrophages and neutrophils, which secrete mediators that promote the sensitization of such nociceptors [76]. These include pro-inflammatory cytokines such as TNF-α, IL-1β, IL-6, and IL-17, nerve growth factor (NGF), serotonin, histamine, and prostaglandin E2 (PGE2), among others [77]. Furthermore, local release of nitric oxide (NO) by inflammatory stimuli is been associated with peripheral nociception. Several studies have suggested PGE2 increased production of PGE2 in the presence of NO, due to the ability of NO to activate COX-1 and up-regulate COX-2, which leads to peripheral release of PGE2 and PGI2 [78]. Sensory neurons express receptors for a number of these mediators, including IL-1βR, TNF-αR, IL-6R, and IL-17RA, NGF receptors (TrkA), and G protein-coupled receptors (GPCR) for serotonin, histamine, and PGE2 [77,79,80,81]. These are expressed in both type C and Aδ nociceptive fibers [82], and their activation enhances membrane excitability, leading to subsequent stimulation due to the hyperactivation of key transduction molecules, such as transient receptor potential vanilloid subtype 1 (TRPV1) and ankyrin subtype 1 (TRPA1) ion channels, and conduction molecules such as tetrodotoxin-insensitive voltage-gated sodium channels Nav1.7, 1.8, and 1.9. This process results in PS, with decreased activation thresholds in primary nociceptors [83]; and in turn, it augments the release of excitatory neurotransmitters at terminal synapses within the dorsal horn (DH) of the spinal cord, a key site for sensory signal modulation. This hyperactivation further sensitizes second-order sensory neurons, triggering neuronal plasticity and CS, a circumscribed state of hyperexcitability in the central nervous system with enhanced processing of nociceptive signals (Figure 2) [50].

Following the development of CS, nociceptive neurons can potently signal to higher-order structures in the brain and brainstem, resulting in pathological pain perception. Furthermore, expansion of receptive fields is often observed, resulting in pain perception from stimulation of uninjured tissue, a phenomenon termed secondary hyperalgesia [84].

Numerous neurobiological mechanisms have been identified in this scenario. Following intense stimulation or persistent injury, activated C and Aδ fibers release a variety of neurotransmitters, including glutamate, substance P, calcitonin gene-related peptide (CGRP), and ATP, onto output neurons of the superficial DH. In consequence, NMDA glutamate receptors (NMDAR) in postsynaptic neurons are activated, increasing intracellular calcium levels and activating a host of calcium-dependent signaling pathways and second messengers, such as mitogen-activated protein kinase (MAPK), protein kinase C (PKC), PKA, and Src [50]. In addition, the increase of the intracellular levels of calcium triggers a cascade of events that include activation of neuronal NO synthase (Nnos), followed by the increase of NO production. The subsequent activation of intracellular receptor soluble guanylyl cyclase (sGC), leads to the activation of the NO-cGMP signaling pathway, present in neurons of the spinal cord implicated in CS, secondary hyperalgesia, and modulation of ion channels. This cascade of events heightens the excitability of output neurons and facilitates the transmission of nociceptive signals to the brain [85]. 

SPMs regulate this aspect chiefly by interacting with TRPV1 and TRPA1 receptors [79,86,87,88]. Low-dose administration of MaR1 and PD1 can reduce TRPV1 activity in neurons in the dorsal roots ganglia (DRG), with their subsequent inhibition caused by PKA and ERK activity [86,87,89,90,91,92,93]. PD1 also decreases hyperexcitability by negatively regulating synaptic transmission induced by TNF-α and LTP in the spinal cord [86,93]. Moreover, RvD1, RvE1, RvD2, and ATL are also powerful TRPA1 and TRPV1 inhibitors [94]; and RvD1 can also suppress TRPV3 and TRPV4 activity [94,95,96,97]. These effects appear to converge in decreased glutamate release in presynaptic neurons, along with decreased ERK phosphorylation, NF-κB translocation, and TNF-α and IL-1β expression [79,94,96,98]. TRPV1 are colocalized with ChemP23 receptors in DRG neurons, which can decrease the hypersensitivity related to mechanical and thermal pain in advanced stages of inflammatory processes by binding RvD1 and RvE1 [79,99]. Similarly, AT-RvD1, another type of Rv, increases ChemR23 mRNA expression [100]. Rv may also modulate NMDAR involved in CS and LTP [89,96].

Indeed, SPMs can modulate the activity of glutamate and NMDAR, which have been linked to hyperalgesia, neuropathic pain, and decreased opioid activity [101,102,103]. In particular, RvE1 can inhibit glutamate release in presynaptic terminals, and consequently reduce NMDAR-mediated potentiation by blocking phosphorylation in the ERK pathway. Importantly, this potentiation is associated with the development of changes in the amplitude and frequency of spontaneous excitatory postsynaptic currents (sEPCs), a neural phenomenon implicated in the processes underlying the neuroplasticity associated with CS and LTP. Therefore, decreasing glutamatergic activity may be a potential mechanism for reducing CP [79,98]. Likewise, RvD1 can prevent the phosphorylation of NMDAR and reduce the expression of proinflammatory cytokines, ameliorating allodynia [104].

Similarly, it has been demonstrated that when the μ opioid receptor is blocked, antinociception of RVD1 and its ligand TRPA1 is prevented. This supports the hypothesis of an imbalance in the endogenous pro and antinociceptive systems, or the TRPA1 and opioid receptors. However, increased research is needed to comprehend this phenomenon [105]. Interestingly, the activation of leukocyte-specific opioid receptors was recently reported to attenuate pain after nerve injury in mice by hyperpolarizing central and peripheral sensory neurons and diminishing the release of excitatory mediators from these neurons, including substance P [106,107], CGRP [108], and glutamate [109]. Indeed, convincing evidence delineates analgesic effects for opioid peptides derived from immune cells in both animal and human models [110,111,112,113].

Other receptors bear important implications in the resolution of inflammation and pain, including GPR37, GPR18, and GPR32. RvD1, RvD3, and RvD5 are ligands for the latter, whose inhibition is related to analgesic properties that could be sex-dependent [114,115]. On the other hand, activation of GPR37 by PD1 modulates macrophage-mediated phagocytosis by increasing intracellular calcium mobilization related to signal transduction mediated by Gi/o subunits, ERK and PI3K/AKT, all involved in the resolution of pro-inflammatory and neuropathic pain states [74,93,116,117]. Furthermore, PD1/GPR37 interactions potentiate the activity of M2 macrophages, in association with the release of β-endorphins [118,119]. On the other hand, GPR18 is a cannabinoid receptor whose ligand is RvD2, which, when activated, increases cAMP levels without changes in intracellular calcium traffic, downregulating various components related to CS such as TRP, MAPK, and JNK phosphorylation, as well as NMDAR [52,120]. Finally, LXA4 also acts as a positive allosteric modulator in CB1 receptors, reducing neuronal excitability [121]. It has been hypothesized that CB1 modulation could restore the levels of IL-1β and COX2 after inflammatory stimuli [122]. 

Glial cells play a key role as mediators for the neuroactive effects of SPMs [123,124]. Both microglia and astrocytes express specific receptors for SMP such as ALX/FPR2, which can inhibit phosphorylation and activation of MAPK such as p-p38, ERK, and JNK [125,126,127,128,129,130]. LX activity has been linked with increased SOCS-1 mRNA levels, which is related to decreased synthesis of pro-inflammatory cytokines induced by JAK-STAT [131,132]. Likewise, MaR1, PD1, RvD1, and RvD2 appear to reduce inflammatory pain by inactivating microglia and astrocytes through similar mechanisms, in addition to inhibiting nuclear translocation of NF-κB and the subsequent release of TNF-α, IL-1β, and IL-6 in peripheral and central nociceptor neurons [131,132]. In turn, both microglia and astrocytes express GRP18 cannabinoid receptors. Their activation by RvD2 may be related to PI3K/AKT/GSK-3β pathway inactivation, triggering inhibition of NF-κB and the release of anti-inflammatory substances such as TGF-β1 in the spinal cord [133]. On the other hand, PD1, LXA4, and MaR1 can decrease the expression of IBA-1 and P2Y12 in microglia and GFAP in astrocytes, with amelioration of mechanical allodynia and thermal hyperalgesia [14,134,135].

Moreover, IL-1β also appears to promote BDNF release in enteric glial cells in subjects with irritable bowel syndrome [136]; and levels of this cytokine have also been correlated with the severity and frequency of abdominal pain in this context [137]. The contribution of astrocytes to CS is less clear [10,77]; although they are also activated after spinal cord injury. This process is typically delayed, yet more persistent, and it can last for up to several months. Thus, astrocytes may be more critical to the maintenance, rather than the induction of CS and persistent pain [77]. 

Likewise, ATP and the chemokine fractalkine (CXCL1) released from nociceptive fibers may also contribute to CS, through the stimulation of spinal microglia. ATP mainly targets microglial P2-type purinergic receptors, triggering the release of brain-derived neurotrophic factor (BDNF). In turn, this activates TrkB in output receptors in lamina I neurons at the DH—a process facilitated by IFN-γ in rodents [138]—which markedly changes chloride ion traffic in these neurons, facilitating depolarization, and thus, increasing excitability and decreasing the response threshold to both noxious and innocuous stimuli [139]. Activation of fractalkine receptor CX3CR1 and Toll-like receptors in microglia also induces the release of BDNF and various cytokines in the spinal cord, favoring CS [140,141].

Oxidative stress, an essential characteristic of neuroinflammatory states, is also targeted by SPMs. LXA4 can increase Nrf2 expression and heme oxygenase translocation to the nucleus [142]; while ATL inhibit the translocation of p47phox to the cell membrane, resulting in lower NADPH oxidase activity and decreased ROS formation by BV2 microglia [143]. In these cells, RvE1 and RvE2 can compete with chemerin for ChemR23 receptors, attenuating pro-inflammatory activity [79,99,116,144]. Likewise, LXA4 may increase superoxide dismutase activity [145]; similarly to MaR1 and RvD1, which appear to upregulate glutathione peroxidase [146]. 

On the other hand, PD1 can suppress the activity of the transcription activating factor 3 (ATF3), which is associated with axonal lesions in DRG and the development of neuropathies [134,147]. In addition, MaR1 exerts neuroprotective effects against oxidative stress by inducing the expression of SOD1 G93AA315T and TDP-43A315T [148]. Lastly, 17-oxoDHA, a metabolite of 17-HDHA, acts as an agonist of peroxisome proliferator-activated receptors (PPAR) α and γ, which appear to have anti-nociceptive effects by lowering LTB4 levels, augmenting LXA4 synthesis and facilitating nitric oxide activity [11,149,150].

### 3.3. Preclinical and Clinical Evidence on Specialized Pro-Resolving Lipids Mediators in the Management of Pain 

In light of the many mechanistic links between SPMs and the pathophysiology of CP, their potential for clinical use has become a key emergent object of research. Preclinical data underlines the efficacy of LX in various animal models of pain, including chronic post-ischemic pain (CPIP) [151], carrageenan-elicited pain (CEP) [127,152], bone cancer-induced pain (BCIP) [130], spinal cord injury (SCI) [15,129,153], chronic constriction injury (CCI) [154], chronic compression of the dorsal root ganglion (CCD) [155], and non-compressive lumbar disc herniation [128].

Notably, in a recent study by Liu et al., BML-111—an LXA4 receptor agonist—was used in rats with SCI, resulting in significantly lower levels of TNF-α, IL-1β, and IL-6 in serum and spinal cord tissue [129]. Sun et al. reported similar results in CCD models, where LXA4 reduced mechanical hypersensitivity in association with decreased TNF-α, IL1β, and IL-6 [155]. Treatment with LXA4 and LXB4 has also been described to reduce mechanical allodynia in rats with BCIP [130]; and LXA4, LXB4, and AT-LXA4 appear to reduce mechanical hypersensitivity in carrageenan-treated rats [127,152]. Besides, treatment with AT-LXA4 has been reported to decrease chronic morphine-induced thermal hyperalgesia by blocking NALP1-derived IL-1β levels in vivo and in vitro, pinpointing a promising pharmacological target for the treatment of pain [156].

Regarding SPMs derived from DPA and DHA, many studies have shown efficacy for RvD, PD, and MaR in animal models of pain, such as CCI [134], formalin-induced pain (FIP) [79,93,94,96], capsaicin-induced pain [89,93,94], complete Freund’s adjuvant (CFA)-induced pain [79,93,94,96,105], osteoarthritis pain [95], and CEP pain [79,93,100,152]. They may also intervene significantly in temporomandibular joint inflammatory pain [92], CPIP [151], chronic post-thoracotomy pain (CPTP) [120,157], post-operative pain induced by tibial bone fracture (PTBF) [133], herniation-induced radicular pain [158], chronic pancreatitis-induced visceral pain [104], and peripheral neuropathic pain induced by chemotherapy (PNPC) [114].

A study performed by Zhang et al. evaluated intravenous perioperative treatment with DHA (500 μg), RvD1 (500 ng), and MaR1 (500 ng) on a mouse model of PNPC, revealing efficacy for these alternatives in pain prevention and delay. They may also alleviate established pain, though this remains less clear [159]. Likewise, RvD2 has been reported to reduce heat hyperalgesia, mechanical allodynia, second-phase spontaneous pain, and thermal sensitization in FIP, CEP, capsaicin-induced pain, AITC, CFA, and fibromyalgia-like pain [94,160]. Conversely, Luo et al. did not find analgesic effects with intrathecal administration of RvD3 and RvD and reported a reduction in mechanical hyperalgesia with RvD5 treatment only in CIPN male mice [114].

Finally, RvE has also been found effective in animal models of CFA-induced inflammatory pain [79,161], FIP [37,161], CEP [79,161], CCI [123], and spinal nerve injury (SNI) [123]. Indeed, in an equivalent dose 1000 times lower than morphine, RvE1 appears to reduce second-phase spontaneous pain in FIP models [79]. Furthermore, in two studies, Xu et al. reported a decrease in mechanical allodynia, heat hyperalgesia, edema, neutrophil infiltration, and expression of pro-inflammatory cytokines and chemokines in diverse pain models treated with RvE1 [79,123]. This is notable, since RvE1 has been shown to be significantly increased by n-3 fatty acids supplementation in humans [162]. Even though effective in reducing inflammation, no robust analgesic effects have been described for RvE2 and RvE3 [163,164]. On the other hand, a phase 2 study examined the therapeutic effectivity of RX-10045, a synthetic analog of RvE1, in the treatment of eye inflammation and pain in cataract surgery [165].

Beyond mechanistic and animal studies, SPMs have also been tested as analgesics in humans with various pain-related conditions (Table 1), such as chronic headaches [166], migraines [167], joint discomfort [15,168], sickle cell disease [169], diabetic neuropathy [170], and various rheumatic diseases [15,171,172,173,174,175]. Firstly, supplementation with certain doses of ω-3 PUFAs appears to produce an increase in circulating anti-inflammatory mediators in humans [176]. For example, in a meta-analysis by Goldberg et al. [15] included 17 trials evaluating the pain-relieving effects of ω-3 PUFA in patients with rheumatoid arthritis or joint pain related to inflammatory bowel disease and dysmenorrhea. The researchers found that supplementation reduced patient-reported joint pain intensity (SMD: −0.26; 95% CI: –0.49 to −0.03, *p* = 0.03), minutes of morning stiffness (SMD: −0.43; 95% CI: −0.72 to −0.15, *p* = 0.003), number of painful and/or tender joints (SMD: −0.29; 95% CI: −0.48 to −0.10, *p* = 0.003), and NSAID use (SMD: −0.40; 95% CI: −0.72 to −0.08, *p* = 0.01). 

Likewise, a 12-month, double-blind, controlled study compared daily supplementation with various doses of ω-3 PUFA, reporting significant improvement in patients’ global evaluation and physicians’ assessment of pain in those taking doses of 2.6 mg/day. This group also significantly reduced the use of anti-rheumatic medications [173]. Similarly, in a randomized, double-blind, placebo-controlled trial fish oil with ω-3 PUFA reduced symptoms of pain and stiffness significantly after nine weeks of treatment were observed [168]. In the “En Balance-Plus” study, an interventional study designed to assess the efficacy of dietary ω-3 PUFA in diabetic patients, significant reductions in pain-related neuropathy symptoms were reported after three months [170]. A randomized, parallel-group, 12-weeks trial with a diet high in ω-3 fatty acids and low in ω-6 PUFA demonstrated a reduction in pain frequency, intensity, and psychological distress in patients with chronic headaches [166]. Various other clinical trials have evaluated the use of PUFA in conditions such as pediatric sickle-cell anemia [177], post-traumatic headache [178], fibromyalgia [179], among others [180,181,182], with promising results. Although these effects may not be wholly attributed to SPM—with changes in sphingolipid metabolism also playing a purportedly significant part—their significance in this context is notable.

## 4. Conclusions

Currently, CP remains one of the most prevalent clinical entities in clinical medicine, related to a wide range of diseases. In response, in recent years there has been a search for novel alternatives for its management. SPMs stand out in this scenario as a group of bioactive lipids which play a fundamental role in the resolution of inflammation and can ameliorate CP through various mechanisms.

These molecules have immunomodulatory properties which can diminish inflammation associated with peripheral and central nociception. Abundant preclinical and clinical evidence supports the role of SPMs in neuroinflammation associated with CS and LTP, either through the modulation of microglia, the regulation of nociceptors, or the regulation of the neuronal pathways implicated in pain.

Nevertheless, further high-quality studies are necessary to better characterize the clinical utility of SPMs in CP, especially attending to the large variety of etiologies, pathophysiologic mechanisms, and clinical presentations that may be associated with this entity. Likewise, deeper research in this area would allow the assessment of adverse effects, tolerability and pharmacological interactions of SPMs. Continued investigation in this field is worthwhile, as SPMs could prove to be an invaluable treatment tool in the future.

## Figures and Tables

**Table 1:** Summary of critical clinical evidence regarding specialized pro-resolving lipid mediators and pain-related conditions.

Authors (REF)	Pain-Related Conditions	Methodology	Results
Goldberg et al. [15]	Rheumatoid arthritis and joint pain	Meta-analysis with 17 randomized controlled clinical trials evaluating the pain-relieving effects of ω-3 PUFA in patients with rheumatoid arthritis or joint pain related to inflammatory bowel disease and dysmenorrhea.	Treatment with ω-3 PUFA for 3–4 months reduced patient-reported joint pain intensity (SMD: −0.26; 95% CI: −0.49 to −0.03, *p* = 0.03), minutes of morning stiffness (SMD: −0.43; 95% CI: −0.72 to −0.15, *p* = 0.003), number of painful and/or tender joints (SMD: −0.29; 95% CI: −0.48 to −0.10, *p* = 0.003), and NSAID use (SMD: −0.40; 95% CI: −0.72 to −0.08, *p* = 0.01).
Geusens et al. [173]	Rheumatoid arthritis	Randomized, double-blind controlled trial that assessed the long-term effects of supplementation with various doses of ω-3 PUFA in 90 patients with active rheumatoid arthritis.	There was a significant improvement in the patient’s global evaluation and the physician’s assessment of pain in patients treated with 2.6 mg/day of ω-3 (*p* < 0.05).
Tajmirriahi et al. [167]	Migraine	Randomized, single-blind clinical trial that evaluated the effect of dietary supplementation with fish oil for migraine prevention in 67 patients taking sodium valproate.	There was a significant decrease in frequency (mean baseline from 13.7 to 2.4; *p* = 0.044), and severity of migraines (mean baseline from 7.9 to 2.9; *p* = 0.046) in participants treated with sodium valproate and fish oil supplementation after the first month of treatment.
Tomer et al. [169]	Sickle cell disease	A double-blind clinical trial that assessed the effects of dietary ω-3 PUFA on the frequency of pain episodes in patients with sickle cell disease in comparison with controls on olive oil.	Treatment with dietary ω-3 PUFA for 1 year reduced the frequency of pain episodes (*p* < 0.01).
Durán et al. [170]	Diabetic neuropathy	Interventional single-group study to assess the efficacy of dietary ω-3 PUFA in 40 participants with type 2 diabetes.	There was a significant reduction in pain-related neuropathy symptoms after three months of treatment with ω-3 PUFA (change from baseline −2.1 (*p* = 0.014) and −9.2 (*p* = 0.002)).
Ramsden et al. [166]	Chronic headache	Randomized, parallel-group and 12-week trial designed to test the clinical effects of a diet high in ω-3 and low in ω-6 PUFA compared to a diet low in ω-6 PUFA in 67 subjects with chronic headaches.	There was reduction of pain frequency (*p* < 0.001), intensity (*p* < 0.001), and psychological distress (*p* = 0.022) in patients treated with a diet high in ω-3 and low in ω-6 PUFA.

Abbreviations: PUFA: polyunsaturated fatty acids; NSAID: non-steroidal anti-inflammatory drugs.

## Data Availability

Not applicable.
